# The Christian Era

**Published:** 1883-08

**Authors:** 


### THE CHRISTIAN ERA.

The much debated question as to the correctness of the hitherto accepted reckoning of the years which have elapsed since the birth of Jesus has again been mooted by Professor Sattler, of Munich, in the columns of a German contemporary. Professor Sattler (according to the *Jewish Chronicle*) claims the distinction of having solved the problem, and of having demonstrated the fact that the current year is probably 1888 instead of 1883. He bases his proofs mainly on three coins which were struck in the reign of Herod Antipas, son of Herod the Great, and which date, consequently, from the first half of the first century of the current era. Madden admits the genuineness of these coins, and other numismatic writers do the same. The evidence they offer coincides with the narrative of the Gospels and with astronomical calculations.

The following are the results at which Professor Sattler has arrived: Jesus was born on the 25th of December, 749 years after the founding of Rome, and commenced his public career on the 17th of November, 780 years after the founding of Rome. He was then 30 years, 10 months and 22 days old. The date on which he commenced his career fell in the 15th year of the Emperor Tiberius, and in the 46th year after the building of Herod's Temple. This is in accordance with St. Luke iii. 1 and St. John ii. 20. According to Josephus ("Antiquities," xv. 11, 1), the construction of Herod's Temple was commenced in the 18th year of that monarch, or in the year 734 after the founding of Rome, in the month of October. If we add the 46 years which elapsed after the building of the Temple, we arrive at the end of the year 780, the year during which Jesus entered on his career. If, moreover, we subtract from 689 (779 years, 10 months and 17 days), 30 years, 10 months and 25 days, there remain 748 years, 11 months and 25 days, which gives us the date of his birth—the 25th of December of the 749th year after the founding of Rome. Jesus died on the 7th of April, 783 of the Roman era; that is to say, on the Friday before Passover, for it has been ascertained by exact calculation that Passover fell that year on the 9th of April, 783; and as the latter year was a Jewish leap year, and consisted, accordingly, of 13 months, the public career lasted two years and seven months. Between the 17th of November, 780, and the 9th of April, 783, three Passovers were celebrated —viz., 781, 782 and 783. Those years correspond with the 27th, 28th, 29th and 30th of the Christian era as at present calculated. Remembering, however, that the year of the birth of Jesus corresponds with the year 749 of the Roman era, and taking this year as the starting point of the Christian reckoning, the years of Jesus' career must be the 31st, 32d, 33d and 34th of the new era. It thus results, according to Professor Sattler, that the Christian reckoning is at fault by five years, and that we are now in 1888 and not in 1883.—*English Mechanic.*

---

To Improve Coal Oil Light.—Add one-eighth to one-fourth amount of common salt. Makes light more brilliant, prevents smoking and keeps wick clean.

Every one in this world has his or her share of troubles and trials. Let us, then, try as much as we are able not to increase the burden of any by as much as the weight of a straw.

Heard in the suburbs: "Are you going to keep your brickyard running this season?" "No; I think I will put a bay window in the kiln, and advertise for summer boarders."—*Phila. News.*

Enemies of the Telegraph.—There is, apparently, no apparatus so liable to be interfered with by what we may call natural causes as the electric telegraph. Fish gnaw and mollusks overweight the submarine conductors on the subterranean wires; while there is at least one instance of a gay and frolicksome whale entangling himself in a deep sea cable, to his own astonishment, and the cable's utter disorganization. It is stated that within three years there have been sixty serious interruptions to telegraphic communications in Sumatra by those seriously curious animals—elephants. In one instance, these sagacious creatures, not thoroughly comprehending the singular contrivance, but most likely fearing snares, destroyed a considerable portion of the line, hiding away the wires and insulators in a canebrake. Monkeys of all tribes and sizes, too, use the poles and wires as an improved gymnasia; while the numerous tigers, bears and buffalos render the watching and repair of the line a duty of great danger.

